# Medical student selection process enhanced by improving selection algorithms and changing the focus of interviews in Australia: a descriptive study

**DOI:** 10.3352/jeehp.2022.19.31

**Published:** 2022-11-28

**Authors:** Boaz Shulruf, Gary Mayer Velan, Sean Edward Kennedy

**Affiliations:** Office of Medical and Health Education, Faculty of Medicine and Health, The University of New South Wales, Sydney, NSW, Australia; Hallym University, Korea

**Keywords:** Algorithms, Consensus, Linear models, Medical schools, School admission criteria

## Abstract

**Purpose:**

The study investigates the efficacy of new features introduced to the selection process for medical school at the University of New South Wales, Australia: (1) considering the relative ranks rather than scores of the Undergraduate Medicine and Health Sciences Admission Test and Australian Tertiary Admission Rank; (2) structured interview focusing on interpersonal interaction and concerns should the applicants become students; and (3) embracing interviewers’ diverse perspectives.

**Methods:**

Data from 5 cohorts of students were analyzed, comparing outcomes of the second year in the medicine program of 4 cohorts of the old selection process and 1 of the new process. The main analysis comprised multiple linear regression models for predicting academic, clinical, and professional outcomes, by section tools and demographic variables.

**Results:**

Selection interview marks from the new interview (512 applicants, 2 interviewers each) were analyzed for inter-rater reliability, which identified a high level of agreement (kappa=0.639). No such analysis was possible for the old interview since it required interviewers to reach a consensus. Multivariate linear regression models utilizing outcomes for 5 cohorts (N=905) revealed that the new selection process was much more effective in predicting academic and clinical achievement in the program (R^2^=9.4%–17.8% vs. R^2^=1.5%–8.4%).

**Conclusion:**

The results suggest that the medical student selection process can be significantly enhanced by employing a non-compensatory selection algorithm; and using a structured interview focusing on interpersonal interaction and concerns should the applicants become students; as well as embracing interviewers’ diverse perspectives.

## Introduction

### Background/rationale

Selecting medical students is the most important assessment undertaken in any medical school. For example, each year, only 130 out of about 3,000 local applicants (4.3%) are offered a place at the Medical School at the University of New South Wales, Australia (UNSW). Once admitted to the medicine program, 94.3% of students graduate as qualified doctors in Ireland excluding transferred students to other medical courses [1]. This harsh reality makes medicine one of the most competitive academic programs worldwide. Medical schools are constantly under pressure from all stakeholders to ensure that their selection processes are robust, fair, and non-discriminatory [2]. Research into the quality of medical student selection is rich [3]. Nonetheless, published research into the effectiveness of traditional versus revised selection processes is mostly limited to the impact of the introduction or omission of a single tool used within a battery of selection tools [4].

The current study conceived from the need to design a purposely tailored selection process to address the overarching objectives of the Faculty’s Selection and Re-admission Committee: selecting applicants who are most suitable for the medicine program; minimizing the number of admitted students who are not suitable; and minimizing the impact of social-demographic biases on the selection outcomes.

Before the design of the new selection process research was undertaken to understand the effectiveness of the tools already used for the selection of UNSW medicine students. The research identified that previous academic achievement measured by the Australian Tertiary Admission Rank (ATAR) has been the best predictor for academic achievement in Australian and New Zealand medical schools [3]. Furthermore, scores in the Undergraduate Medicine and Health Sciences Admission Test (UMAT) correlated positively with academic achievements in the medicine program, but to a lesser extent than ATAR [3,5]. On the other hand, the selection interview which had been used at UNSW since 2004, was ineffective at predicting outcomes in the program [6].

As a result, a new selection process was designed in 2017 (for the incoming 2018 cohort), based on the following principles:

First, the selection tools must be based on the best available evidence from research in relevant fields; second, the selection process needs to focus primarily on identifying concerns rather than strengths, since the number of applicants who have significant strengths across the domains assessed far exceeds the number of places available in the program. It is noteworthy that students who struggle or fail in the medicine program are among those who had ranked at the top 8% of the applicants in their cohort. Thus, had concerns been identified earlier, more suitable candidates might have been selected instead; third, the selection should minimize bias against applicants based on their socio-demographic background.

The ATAR marks used for selection include adjustments to accommodate disadvantages due to socio-demographic factors and disability, thus this tool continued to be used in the new selection process. The adjusted ATAR is termed “Universities Admissions Centre (UAC) Rank” and henceforth the term UAC Rank will be used. Similarly, the UMAT continued to be used as the most widely available medical aptitude test in Australia, with an established evidence base [5]. However, the selection interview had been completely changed from a semi-structured interview, which aimed to identify strengths and motivation to study medicine, to a structured interview aimed to elicit potential concerns. The domains of the new interview were determined by faculty academic and clinical staff from a list of specific attributes regarded as important for medical students and doctors; as well as from the literature on the psychology of decision making [7], and design of interview questions [8].

The assumption was that every successful applicant should be able to quickly establish good rapport with any person, should be perceived as trustworthy and reliable, and is expected to effectively interact verbally and non-verbally. The difference between old and new admission processes was described in Supplement 1.

The new selection process’s algorithm utilizes a non-compensatory approach [9], whereby performance in each of the 3 selection tools is ranked and the lowest rank among the 3 tools, including within the interview, takes priority for establishing the final selection rank. Thus, a high rank in one tool (e.g., UAC Rank) cannot compensate for a lower rank in another (e.g., interview). Applicants need to perform well across all selection tools to be offered a place in the medicine program [9].

### Objectives

The purpose of this study was to compare the efficacy of the new medicine selection process introduced for the incoming cohort in 2018 and the old selection process to estimate the students’ competency. This report comprises the results of a series of analyses of selection and assessment data for the 2014–2018 incoming cohorts. The 2014–2017 cohorts were admitted by the old selection process, whereas the 2018 cohort was admitted by the new selection process. The performance of the 2 selection processes in predicting critical assessment outcomes of students completing phase 1 (years 1 and 2) of their medical program is the focus of this study. Although this study uses data from one medical school, its significance is much broader. Identifying “critical features” that can improve the student selection process, in general, may benefit other institutions and other health professions’ education programs.

## Methods

### Ethics statement

This study was approved by the UNSW Human Research Ethics Advisory Panel (ref: HC210373). No informed consent was obtained because it is the analysis of the data of the admission process. Data provided for the analysis had been standardized to maintain student anonymity and confidentiality as per ethical requirements. For the new interview inter-rater reliability analysis, the data consisted of the interviewers’ marks only.

### Study design

It is a descriptive study using administrative data for student selection of 5 cohorts of students (2014–2018), including one cohort for a new process and the other 4 cohorts for an old process. It was described according to STROBE Statement available from: https://www.strobe-statement.org/.

### Setting

Data used for this study were obtained from the administrative data repository of the UNSW Faculty of Medicine & Health. Assessment outcomes of interest were 3 ends of phase 1 assessment outcomes, which are assessed at the end of year 2 in the medicine program (phase 1 clinical and communications skills examination, phase 1 end-of-phase examination, phase 1 portfolio examination). These data pertained to all applicants who secured a place and studied in the program. These data were made available for analysis after all assessment scores were standardized (mean=50; standard deviation [SD]=5) to maintain student anonymity. In addition, for assessing the inter-rater reliability of the new selection interview, marks for all applicants who were interviewed for selection in 2018 were used, irrespective of whether the applicants were admitted to the program.

### Participants

The data used comprised selection tool marks of applicants who applied to the medicine program at UNSW in the years 2014–2018. All applicants applied for domestic places either by the general or the rural admission pathway.

### Variables

Variables included in the dataset are as follows: gender; selection process by cohorts (old selection process, 2014–2017 or new selection process, 2018); selection pathway (metropolitan or rural); UAC Rank, which is the standardized secondary school total assessment score derived from the ATAR; socioeconomic status decile derived from the applicant’s reported home postcode; Interview Standardized Score; and UMAT standardized scores assessment outcomes: phase 1 (end of year 2) standardized marks of portfolio exam, clinical & communications skills exam, end of year written exam.

### Data sources/measurement

All anonymized data were obtained from the administrative data repository of the UNSW Faculty of Medicine & Health (Dataset 1). There was no measurement tool used.

### Bias

No known biases in the data were identified.

### Study size

This study used all the available data from 5 cohorts (N=905). To identify medium effects the minimum sample size required for 6 predictors of the regression analysis is 97 [10].

### Statistical methods

Inter-rater reliability of interviewers’ judgments (in total 512 interviews) was analyzed for the 2018 incoming cohort. The data were analyzed under 2 different assumptions regarding judgment data: first, the data comprised 4 discrete categories, thus kappa statistics were used; second, the alternative assumption was that the judgment data comprised of an interval 1–4 scale, in which case intraclass correlation coefficient (ICC) was used together with Cronbach’s α. The 2018 data were also analyzed to identify the impact of gender on the selection tools, including the interview and the final selection rank, using independent t-tests. Since the selection algorithm generates a rank rather than a score, that rank was normalized to a score between 0–100 (mean=50, SD=12.9). It was not possible to compare inter-rater reliability for the old interview since the data included only the score agreed by consensus between the 2 interviewers. The second part of the analysis consists of a series of linear regressions models each model predicts one of the 3 outcomes (phase 1 clinical and communications skills examination, phase 1 end-of-phase examination, phase 1 portfolio examination) by the following predictors: gender; socioeconomic status; selection pathway (rural/non-rural); UAC Rank; mean UMAT score; interview score (for 2014–2017 cohorts the old interview score, for the 2018 cohort the new interview score).

## Results

### Participants

Participants’ mean age was 18.9 years and 895 out of 905 (99%) of interviewees were between 17–22 years of age. Men comprised 47.1% of the population. The distribution of student population by gender did not differ significantly across selection years (chi-square=5.524, P=0.238) but overall, there were slightly more women (52.9%) than men (47.1%) (Table 1). Students’ UAC Rank, UMAT, and socioeconomic status also did not have any statistical difference across cohorts (Table 2).

### Main results

#### Quality of the new interview

**Fig. 1.**
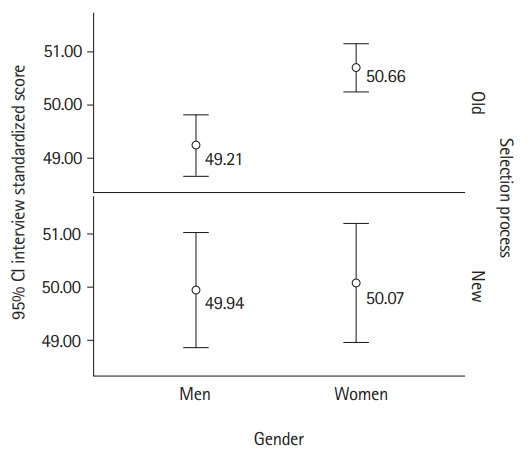
Interview scores by gender by selection process. CI, confidence interval.

Data from 512 interviews were available for this analysis. The results demonstrated a good level of inter-rater reliability across the 3 statistics: kappa=0.639; ICC=0.895 (absolute agreement); and Cronbach’s α=0.895 (P<0.001 for all these measures). Demographic data, which was available only for the interviewees who were offered a place in the program, demonstrated that the new interview performed better in terms of gender balance (Fig. 1) and no impact of socioeconomic status interview scores was identified for either the old or the new interview (r=-0.041, P>0.05 and r=-0.043, P>0.05, respectively).

#### Comparison of predictability of the students’ performance selected by between old selection process and new selection process

**Fig. 2.**
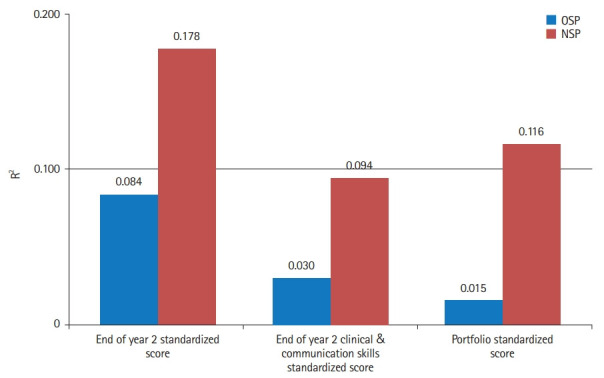
Total variance explained (%) in the regression models. OSP, old selection process; NSP, new selection process.

The focus of this study comprises a series of 6 linear regression models estimating the predictability of the 3 key assessment outcomes of phase 1 of the medicine program by the old and new selection processes (Tables 3–5). These results suggest that the new selection process is superior to the old selection process both according to the impact of individual selection tools, as well as by overall impact. Using the variance explained (R^2^) as a measure of model efficacy, the new selection process yielded R^2^ 3.13 times higher than the old selection process for predicting phase 1 clinical and communications skills examination outcomes (Table 3, Fig. 2), 2.11 times higher for predicting phase 1 end-of-phase examination marks (Table 4, Fig. 2), and 7.73 times higher for predicting phase 1 portfolio examination results (Table 5, Fig. 2). Comparing the impact of the new and old interviews on these outcomes, demonstrates that the old interview had a negative impact on predicting end of phase 1 examination results and no significant impact on other outcomes (Table 3), whereas the new interview had a significant positive predictive impact on phase 1 clinical and communications skills examination results (Table 4). Indeed, this was the largest impact among all other predictors for that outcome (beta=0.175, P=0.023) (Table 4). This pattern of the new selection process having a higher impact compared to the old selection process is consistently shown across all 3 selection tools for all 3 outcomes, except for the UMAT scores which have a negligible impact on the portfolio scores in both selection processes (Table 4).

## Discussion

### Key results

Overall, the results of this study suggest that the new selection process is superior to the old selection process on several accounts. The predictability of all 3 critical outcomes by the new selection process was 2.1–7.7 times higher than the old selection process (Fig. 2), and the new selection process outcomes were not significantly impacted by demographic variables.

### Interpretation

The main question is why the new selection process is so much better than the old selection process? It is suggested that the new selection process algorithm plays a key role in its superiority. A recent study that modeled a non-compensatory model for the selection of applicants to interview for medicine at UNSW demonstrated that a non-compensatory model raises the selection threshold for both ATAR and UMAT without significantly impacting the mean socioeconomic status score of the selected group (Table 4) [9]. In practice, the new selection process is more likely to de-select applicants who ranked low on one selection tool despite being ranked highly on the other 2 tools. Such applicants were likely to be selected due to the compensatory nature of the old selection process. A non-compensatory selection model takes into account potential concerns because a low rank on any selection tool would ultimately have a greater impact on the chance of being offered a place in the medicine program than in the previous compensatory model. The merit of this concept has already been demonstrated, showing that thresholds can be determined in selection tool scores that predict success in the medicine program. In some instances, these thresholds yielded high (up to 1.20) effect sizes [3].

Since the non-compensatory model did not negatively impact the socioeconomic status distribution of the selected cohorts, it is suggested that medical schools consider adopting such an approach, irrespective of the battery of selection tools employed.

The efficacy of the new selection process compared to the old selection process is also notable. In the old selection process, UAC Rank and UMAT had a relatively small predictive impact on phase 1 end-of-phase examination outcomes (beta=0.145, P=0.003 and beta=0.039, P=0.345, respectively), whereas the new selection process using the same tools is a much better predictor of outcomes (beta=0.351, P<0.001 and beta=0.139, P=0.136, respectively). Also, the overall variance explained by the regression model was much higher for the new selection process (R^2^=17.8%) compared with the old selection process (R^2^=8.4%). Since the ATAR, UMAT and student demographic characteristics had not changed across the 2014–2018 cohorts, the most plausible reason for the change in the efficacy of the models is the selection algorithm.

The new interview also plays an important role in the new selection process’ superiority over the old selection process as can be seen in the regression models (Table 3). The new interview had the largest impact on phase 1 clinical and communications skills outcomes (beta=0.175, P=0.023) among all predictors. By comparison, the old interview had a negligible impact (beta=0.026, P=0.527) (Table 3). This demonstrates that the selection interview is relevant to an important set of clinical skills that are required in the medicine program. Moreover, when it comes to predicting outcomes of phase 1 end-of-phase examination, which represents academic performance, the new interview had no significant impact on that outcome (yet in comparison, the old interview was negatively associated with that outcome: beta=-0.116, P=0.004). Adding to this the good inter-rater reliability yielded by the new interview (kappa=0.639; ICC=0.895; Cronbach’s α=0.895), provides supporting evidence for the validity of the selection interview as it is within the top range of inter-rater reliability reported for such interviews [11]. Thus, the interview is reliable, it demonstrates predictive validity for a relevant outcome and it is not associated with irrelevant outcomes.

When looking at the third outcome, phase 1 portfolio examination (Table 5), the difference between the regression models is even more interesting. First, the variance explained by the old selection process was a negligible 1.5%, whereas for the incoming 2018 cohort the variance explained was 11.6%. The portfolio examination outcome is constructed from students’ academic and clinical activities and it emphasizes students’ reflection upon their accomplishments and challenges. The main difference between the new selection process and the old selection process regression models was that for the old selection process the only significant impact on the outcome was gender (beta=0.090, P=0.020). In the new selection process regression model socioeconomic status had the largest impact (beta=0.209, P=0.029) followed by UAC Rank (beta=0.172, P=0.063) and interview (beta=0.118, P=0.121). These results further strengthen support for the utility of the new selection process, yet the single demographic impact shown here requires further investigation to ensure that the new selection process is socio-demographically neutral and does not inadvertently discriminate against applicants.

### Limitations

The most important limitation of this study is that it employs outcomes that are only 2 years into the medicine program. The original intention was to include assessment data from the end of phase 2 (year 3) in the analysis. However, these data were not usable because learning modes, assessments and grading systems were modified due to the coronavirus disease 2019 pandemic in 2020 and 2021. Consequently, those outcomes could not be compared with related outcomes of the cohort selected by the old selection process. Future analysis is planned to further determine the efficacy of the unique features in the new selection process in predicting advanced outcomes in the medicine program.

### Conclusions

This study provides evidence to support the inclusion of important features in the medical student selection process. Using a non-compensatory selection algorithm; using a future-focused interview that aims to identify potential concerns should the applicant become a medical student and later a doctor; and overall making sure the selection process minimizes the selection of less suitable applicants, rather than identifying the top candidates. The top performers will always be selected, but for the benefit of students, educators, and our broader communities, we need to further minimize the number of unsuitable applicants securing a place in health professional programs.

## Figures and Tables

**Table 1.** Student distribution by gender by year at the University of New South Wales

Variable	Selection year					Total
	2014	2015	2016	2017	2018	
Men						
Count	97	77	81	80	91	426
% Within gender	22.8	18.1	19.0	18.8	21.4	100.0
% Within selection year	49.0	42.8	46.0	44.0	53.8	47.1
Women						
Count	101	103	95	102	78	479
% Within gender	21.1	21.5	19.8	21.3	16.3	100.0
% Within selection year	51.0	57.2	54.0	56.0	46.2	52.9
Total						
Count	198	180	176	182	169	905
% Within gender	21.9	19.9	19.4	20.1	18.7	100.0
% Within selection year	100.0	100.0	100.0	100.0	100.0	100.0

**Table 2.** Differences in UAC Rank, UMAT, and SES across cohorts by the analysis of variance across cohorts at the University of New South Wales from 2014 to 2018 cohorts

Variable	Sum of squares	df	Mean square	F-value	Significance
UAC Rank					
Between groups	11.481	4	2.870	0.987	0.414
Within groups	2,618.439	900	2.909		
Total	2,629.921	904			
UMAT mean					
Between groups	215.756	4	53.939	1.741	0.139
Within groups	27,885.126	900	30.983		
Total	28,100.882	904			
SES decile					
Between groups	16.367	4	4.092	0.521	0.720
Within groups	6,929.136	883	7.847		
Total	6,945.503	887			

UAC, Universities Admissions Centre; UMAT, Undergraduate Medicine and Health Sciences Admission Test; SES, socioeconomic status; df, degrees of freedom.

**Table 3.** Predictability of phase 1 end of year 2 standardized score by demographic variables and selection tools by selection cohort (dependent variable: end of year 2 standardized score)

Selection process	Unstand. Coeff		Stand. Coeff	t-value	Significance	95% CI for B	R^2^
	B	Standard error	Beta				
Old selection process							0.084
(Constant)	46.546	5.411		8.602	0.000	35.923 to 57.170	
Gender	-0.277	0.374	-0.028	-0.741	0.459	-1.011 to 0.457	
Socioeconomic decile	0.037	0.078	0.021	0.473	0.636	-0.116 to 0.190	
Selection pathway (local/rural)	-0.967	0.723	-0.085	-1.338	0.181	-2.387 to 0.452	
UAC Rank standardized score	0.147	0.050	0.145	2.950	0.003	0.049 to 0.244	
UMAT standardized score	0.040	0.051	0.039	0.781	0.435	-0.060 to 0.140	
Interview standardized score	-0.115	0.040	-0.116	-2.855	0.004	-0.194 to -0.036	
New selection process							0.178
(Constant)	19.744	7.636		2.586	0.011	4.663 to 34.824	
Gender	-1.469	0.721	-0.147	-2.039	0.043	-2.892 to -0.046	
Socioeconomic decile	0.091	0.172	0.048	0.528	0.598	-0.249 to 0.430	
Selection pathway (local/rural)	1.071	1.291	0.096	0.830	0.408	-1.478 to 3.620	
UAC Rank standardized score	0.351	0.088	0.351	3.972	0.000	0.177 to 0.526	
UMAT standardized score	0.139	0.093	0.139	1.497	0.136	-0.044 to 0.322	
Interview standardized score	0.109	0.073	0.109	1.499	0.136	-0.035 to 0.253	

Unstand. Coeff, unstandardized canonical discriminant function coefficient; Stand. Coeff, standardized canonical discriminant function coefficient; CI, confidence interval; UAC, Universities Admissions Centre; UMAT, Undergraduate Medicine and Health Sciences Admission Test.

**Table 4.** Predictability of end of year 2 clinical and communication skills standardized score by demographic variables and selection tools by selection cohort (dependent variable: end of year 2 clinical and communication skills standardized score)

Selection process	Unstand. Coeff		Stand. Coeff,hr/>	t-value	Significance	95% CI for B	R^2^
	B	Standard error	Beta				
Old selection process							0.030
(Constant)	43.490	5.563		7.818	0.000	32.568 to 54.412	
Gender	1.331	0.384	0.133	3.461	0.001	0.576 to 2.086	
Socioeconomic decile	0.067	0.080	0.037	0.828	0.408	-0.091 to 0.224	
Selection pathway (local/rural)	1.451	0.744	0.128	1.952	0.051	-0.009 to 2.911	
UAC Rank standardized score	0.080	0.051	0.079	1.572	0.117	-0.020 to 0.181	
UMAT standardized score	-0.009	0.052	-0.009	-0.174	0.862	-0.112 to 0.094	
Interview standardized score	0.026	0.041	0.026	0.632	0.527	-0.055 to 0.108	
New selection process							0.094
(Constant)	26.628	8.018		3.321	0.001	10.792 to 42.464	
Gender	1.437	0.757	0.144	1.900	0.059	-0.057 to 2.932	
Socioeconomic decile	0.264	0.180	0.140	1.465	0.145	-0.092 to 0.621	
Selection pathway (local/rural)	0.836	1.355	0.075	0.616	0.538	-1.841 to 3.512	
UAC Rank standardized score	0.152	0.093	0.152	1.637	0.104	-0.031 to 0.335	
UMAT standardized score	0.082	0.097	0.082	0.844	0.400	-0.110 to 0.274	
Interview standardized score	0.175	0.077	0.175	2.291	0.023	0.024 to 0.327	

Unstand. Coeff, unstandardized canonical discriminant function coefficient; Stand. Coeff, standardized canonical discriminant function coefficient; CI, confidence interval; UAC, Universities Admissions Centre; UMAT, Undergraduate Medicine and Health Sciences Admission Test.

**Table 5.** Predictability of phase 1 end of year 2 portfolio standardized score by demographic variables and selection tools by selection cohort (dependent variable: portfolio standardized score)

Selection process	Unstand. Coeff		Stand. Coeff	t-value	Significance	95% CI for B	R^2^
	B	Standard error	Beta				
Old selection process							0.015
(Constant)	43.232	5.610		7.706	0.000	32.217 to 54.246	
Gender	0.905	0.388	0.090	2.334	0.020	0.144 to 1.666	
Socioeconomic decile	0.052	0.081	0.029	0.641	0.522	-0.107 to 0.211	
Selection pathway (local/rural)	0.333	0.750	0.029	0.444	0.657	-1.139 to 1.805	
UAC Rank standardized score	0.096	0.052	0.095	1.865	0.063	-0.005 to 0.197	
UMAT standardized score	0.026	0.053	0.025	0.487	0.626	-0.078 to 0.130	
Interview standardized score	-0.006	0.042	-0.006	-0.138	0.890	-0.088 to 0.076	
New selection process							0.116
(Constant)	34.655	7.919		4.376	0.000	19.015 to 50.296	
Gender	1.167	0.747	0.117	1.562	0.120	-0.309 to 2.643	
Socioeconomic decile	0.394	0.178	0.209	2.209	0.029	0.042 to 0.746	
Selection pathway (local/rural)	-0.496	1.339	-0.044	-0.371	0.711	-3.140 to 2.148	
UAC Rank standardized score	0.171	0.092	0.172	1.870	0.063	-0.010 to 0.353	
UMAT standardized score	-0.051	0.096	-0.051	-0.529	0.598	-0.241 to 0.139	
Interview standardized score	0.118	0.076	0.118	1.561	0.121	-0.031 to 0.268	

Unstand. Coeff, unstandardized canonical discriminant function coefficient; Stand. Coeff, standardized canonical discriminant function coefficient; CI, confidence interval; UAC, Universities Admissions Centre; UMAT, Undergraduate Medicine and Health Sciences Admission Test.

## Footnotes

**Data availability**
Data files are available from Harvard Dataverse: https://doi.org/10.7910/DVN/K1NLXS Dataset 1. Administrative data for student selection of 5 cohorts of students (2014–2018), including one cohort for a new process and the other 4 cohorts for an old process at the University of New South Wales, Australia.

Dataset 2. Results from the interview of the new selection process including all interviewers’ marks.

## Supplementary materials

Supplementary files are available from Harvard Dataverse: https://doi.org/10.7910/DVN/K1NLXS

Supplement 1. Differences between the old and the new selection interviews at the Medicine Program, University of New South Wales, Australia.

Supplement 2. Audio recording of the abstract.

## References

[1] Maher BM, Hynes H, Sweeney C, Khashan AS, O’Rourke M, Doran K, Harris A, Flynn SO (2013). Medical school attrition-beyond the statistics a ten year retrospective study. BMC Med Educ.

[2] Conrad SS, Addams AN, Young GH (2016). Holistic review in medical school admissions and selection: a strategic, mission-driven response to shifting societal needs. Acad Med.

[3] Shulruf B, Bagg W, Begun M, Hay M, Lichtwark I, Turnock A, Warnecke E, Wilkinson TJ, Poole PJ (2018). The efficacy of medical student selection tools in Australia and New Zealand. Med J Aust.

[4] Griffin B, Horton GL, Lampe L, Shulruf B, Hu W (2021). The change from UMAT to UCAT for undergraduate medical school applicants: impact on selection outcomes. Med J Aust.

[5] Poole P, Shulruf B, Rudland J, Wilkinson T (2012). Comparison of UMAT scores and GPA in prediction of performance in medical school: a national study. Med Educ.

[6] Ma C, Harris P, Cole A, Jones P, Shulruf B (2016). Selection into medicine using interviews and other measures: much remains to be learned. Issues Educ Res [Internet].

[7] Kahneman D, Sibony O, Sunstein C (2021). Noise: a flaw in human judgment.

[8] Willis GB (2015). Analysis of the cognitive interview in questionnaire design.

[9] Shulruf B, O’Sullivan A, Velan G (2020). Selecting top candidates for medical school selection interviews: a non-compensatory approach. BMC Med Educ.

[10] Ryan TP (2013). Sample size determination and power.

[11] Vermeulen MI, Kuyvenhoven MM, Zuithoff NP, van der Graaf Y, Damoiseaux RA (2013). Dutch postgraduate GP selection procedure; reliability of interview assessments. BMC Fam Pract.

