# Supplementary material for: Medical student selection process enhanced by improving selection algorithms and changing the focus of interviews in Australia: a descriptive study
Source: J Educ Eval Health Prof. 2022 Nov 28;19:31. doi: 10.3352/jeehp.2022.19.31 (PMC10435329; doi:10.3352/jeehp.2022.19.31)
Supplement: Supplementary file 3 — Supplement 1. Differences between the old and the new selection interviews at the Medicine Program, University of New South Wales, Australia. [file jeehp-19-31-suppl1.docx]

**Supplement 1.** Differences between the old and the new selection interviews at the Medicine Program, University of New South Wales, Australia

The new selection interview assesses these behaviors and the interviewers’ role is to identify any concerns regarding these behaviors. Moreover, the old interview required interviewers to reach a consensus and report their agreed mark, whereas the new interview requests interviewers to make an independent judgement of each interviewee’s suitability for the program. The other important difference between the old and the new selection interviews is that the old interview’s overarching focus was on the quality of the applicant based on their life experience as of the time of the interview, whereas the new interview considers the extent to which interviewers would like the applicant to be their own doctor sometime in the future. In other words, the focus of judgement was shifted from the applicant’s past to their potential capacity. This also includes a sense of accountability as the outcome of each interviewer’s judgement has potential personal implications for the interviewer.

A summary of the differences between the old selection process (OSP) and the new selection process (NSP) is presented in Table 1. Overall, the old interview focused on the applicants’ life experiences and motivations whereas the new interview focused on applicants’ potential to be safe and effective doctors.

**Table 1.** A comparison between OSP and NSP

| Selection | OSP | NSP |
| --- | --- | --- |
| ATAR/UAC Rank | Used as a score (all ranks were modified to z-scores) | Used as a rank |
| UMAT | Used as a score (scores were modified to z-scores) | Used as a rank |
| Selection interview | Semi structured 40–60 minutes with follow-up questions (scores were modified to z-scores) | Structured 35 minutes, no follow-up questions |
| Domain assessed | Communication skills, motivation, empathy towards others, self-awareness, responding to diversity and ability to cope with uncertainty | Interactions with the community, interactions with people, personal insights, learning experience, motivations |
| Focus of assessment | Retrospective approach- focus on the applicant’s life experiences and their motivation for a life in medicine | Prospective approach- attempt to identify potential concerns should the applicant become a future doctor who would personally look after the interviewer |
| Communication | Focus on communication and language skills | Focus on interpersonal interactions and ability to understand and convey complex information and concepts. |
| Interviewers’ interactions | Interviewers need to reach an agreement | Interviewers do not need to agree, each interviewers’ judgment is established independently and is considered equally. |
| Selection algorithm | A compensatory approach using the average of 3 marks (UAC rank, UMAT, Interview Score) after modified to z-scores; a high score on one tool can compensate for a lower score on another. | A non-compensatory approach, using an algorithm that considers ranks of the 3 marks (UAC rank, UMAT, Interview Score), in which the lowest rank among the 3 tools takes priority for establishing the final selection rank; a high rank on one tool cannot compensate for a lower rank on another. |

OSP, old selection process; NSP, new selection process; ATARm Australian Tertiary Admission Rank; UAC, Universities Admissions Centre; UMAT, Undergraduate Medicine and Health Sciences Admission Test.

There is also a critical difference between the selection algorithms across the OSP and the NSP. The OSP’s algorithm normalized the marks from the 3 selection tools and utilized an average to establish the final selection rank. This follows a compensatory approach, where a high score on one tool can compensate for a low score on another. For example, high academic performance can compensate for poor performance in the interview.
